# Importance of Operational Factors in the Reproducibility of *Aspergillus* Galactomannan Enzyme Immune Assay

**DOI:** 10.1371/journal.pone.0124044

**Published:** 2015-04-10

**Authors:** Nicolas Guigue, Samuel Lardeux, Alexandre Alanio, Samia Hamane, Marc Tabouret, Stéphane Bretagne

**Affiliations:** 1 Laboratoire de Parasitologie-Mycologie, Groupe hospitalier Lariboisière-Saint Louis, Assistance Publique-Hôpitaux de Paris (APHP), Paris, France; 2 Université Paris Diderot, Sorbonne Paris Cité, Paris, France; 3 Bio-Rad Laboratories, Steenvoorde, France; 4 CNRS URA3012, Paris, France; 5 Institut Pasteur, Unité de Mycologie Moléculaire, Centre National de Référence Mycoses Invasives et Antifongiques, Paris, France; Louisiana State University, UNITED STATES

## Abstract

**Background:**

The Platelia *Aspergillus* Ag assay (Bio-Rad) is designed for detecting *Aspergillus* galactomannan (GM) and is widely used for diagnosing invasive aspergillosis but is hampered by variable occurrences of unreproducible positive results. Frequency and origin of these unreproducible results have not been formally studied.

**Methods:**

Different technicians simultaneously performed four tests on 550 consecutive sera from adult patients (Test#1-Test#2 for extraction#1 and Test#3-Test#4 for extraction#2). The samples were classified as confirmed negative [all tests with GM optical density index (GM-ODI) <0.5], confirmed positive (all tests with GM-ODI ≥0.5), extraction unreproducible positive (Test#1 and Test#2 ODIs ≥0.5, and Test#3 and Test#4 GM-ODIs <0.5, or conversely), and ELISA unreproducible positive (only one test with GM-ODI ≥0.5). The samples with positive and negative GM-ODIs within the assay coefficient of variation values were classified as non-conclusive. Four similar additional tests were performed after ≤72h storage at 4°C and a new GM test after 8 months at -20°C.

**Results:**

Five-hundred-twenty sera (94.5%) were confirmed negative, 15 (2.7%) confirmed positive, 4 (0.7%) extraction unreproducible positive, 6 (1.1%) ELISA unreproducible positive, and 5 (0.9%) non-conclusive. Upon retesting, the unreproducible positive results turned negative except for one which turned non-conclusive. The confirmed positive and non-conclusive had similar GM-ODIs (p>0.4) upon retesting after storage ≤72h at 4°C (n = 20) or eight months at -20°C (n = 17).

**Conclusions:**

Operational unreproducible positives represent 33% of the GM-positive results and a second sample evaluation appears mandatory to avoid useless investigations or treatments. When operational artifacts are excluded, GM remains stable at standard storage conditions.

## Introduction

Galactomannan (GM) is a fungal antigen produced by several molds including *Aspergillus fumigatus*. GM release in bloodstream is the rationale for its use for diagnosis [1] and management [2,3] of invasive aspergillosis. The most widely accepted test is the Platelia *Aspergillus* Ag assay (Bio-Rad Laboratories, Marnes la Coquette, France), an Enzyme Linked Immunosorbent Assay using a rat monoclonal antibody [4]. The test is mainly used as a screening test in serum of patients at risk of invasive aspergillosis and has been implemented in numerous hematological wards since the 90’s [5–7]; it is currently accepted as a microbiological criterion for the diagnosis of invasive aspergillosis [8]. Therefore, a GM positive result is often the only element [9], although concerns have been raised about the accuracy of GM testing [1,10].

Performance of the assay is variable, with metaanalysis showing pooled sensitivity of 0.71 and pooled specificity of 0.89 in proven cases of invasive aspergillosis, i.e. based on biopsy diagnosis [11]. False reactivity has always been observed from 5% in adults to as much as 83% in newborn babies [10]. This false positivity rate has led the manufacturer to recommend retesting of positive samples. This resulted in publications underlying the possibility to observe unreproducible positive results [12–20]. Recent reports have also questioned the role of long sample storage in the lack of reproducibility with different conclusions [13,15,16,20–22].

We therefore performed a formal comparison between the routine laboratory diagnosis and a Bio-Rad referent operator who performed additional testing blind to the routine one. Based on this intra-laboratory reproducibility study, our purpose was to characterize the analytical false positive results occurring during testing and to distinguish them from positive results related to the serum and the patient. Among the possible limitations of the GM assay, little attention has been paid to the initial preanalytical preparation steps of the assay as a cause of false GM positivity leading to imprecise or erroneous conclusions in several previous studies.

## Material and Methods

### Ethics Statement

Saint-Louis Hospital is a 650-bed tertiary university hospital with major clinical activities in hematology, renal transplantation and oncology. This study was a non-interventional study with no change in the usual procedures. Biological material and clinical data were obtained only for standard diagnostic following physicians’ prescriptions with no specific sampling. According to the French Health Public Law (CSP Art L1121-1.1), such protocol does not require approval of an ethics committee and is exempted from specific informed consent application.

### GM assay

GM detection was performed by Platelia *Aspergillus* Ag Assay (Ref#62794) following the manufacturer's instructions (Bio-Rad Laboratories). Serum handling was performed under safety cabinet until the heating phase of the procedure. Heating at 100°C was performed in water bath (TW 12, Julabo, Seelbach, Germany). The R3, R4 and R5 reagents are defined as negative control serum, cut-off control serum, and positive control serum, respectively. The optical density (OD) results were translated in GM-OD index (GM-ODI) using the ratio OD-sample/OD-R4 (mean of two replicates). The positivity threshold is GM-ODI ≥0.5 as recommended by the manufacturer. An internal control (mix of different R4 reagents of the same batch since the positive control of the kit cannot be used as interlot control since it is not always calibrated at the same level) was assessed in each series to calculate the inter-assay coefficient of variation (CV). The mean of 104 internal control GM ODIs was 0.94+/-0.14, i.e. a CV of 14.5%. The 95% confidence interval (95% CI) for the 0.5 GM-ODI threshold was 0.36–0.64, after the formula 0.5 +/- 1.96 SD with SD = 0.5 CV.

**Fig 1:**
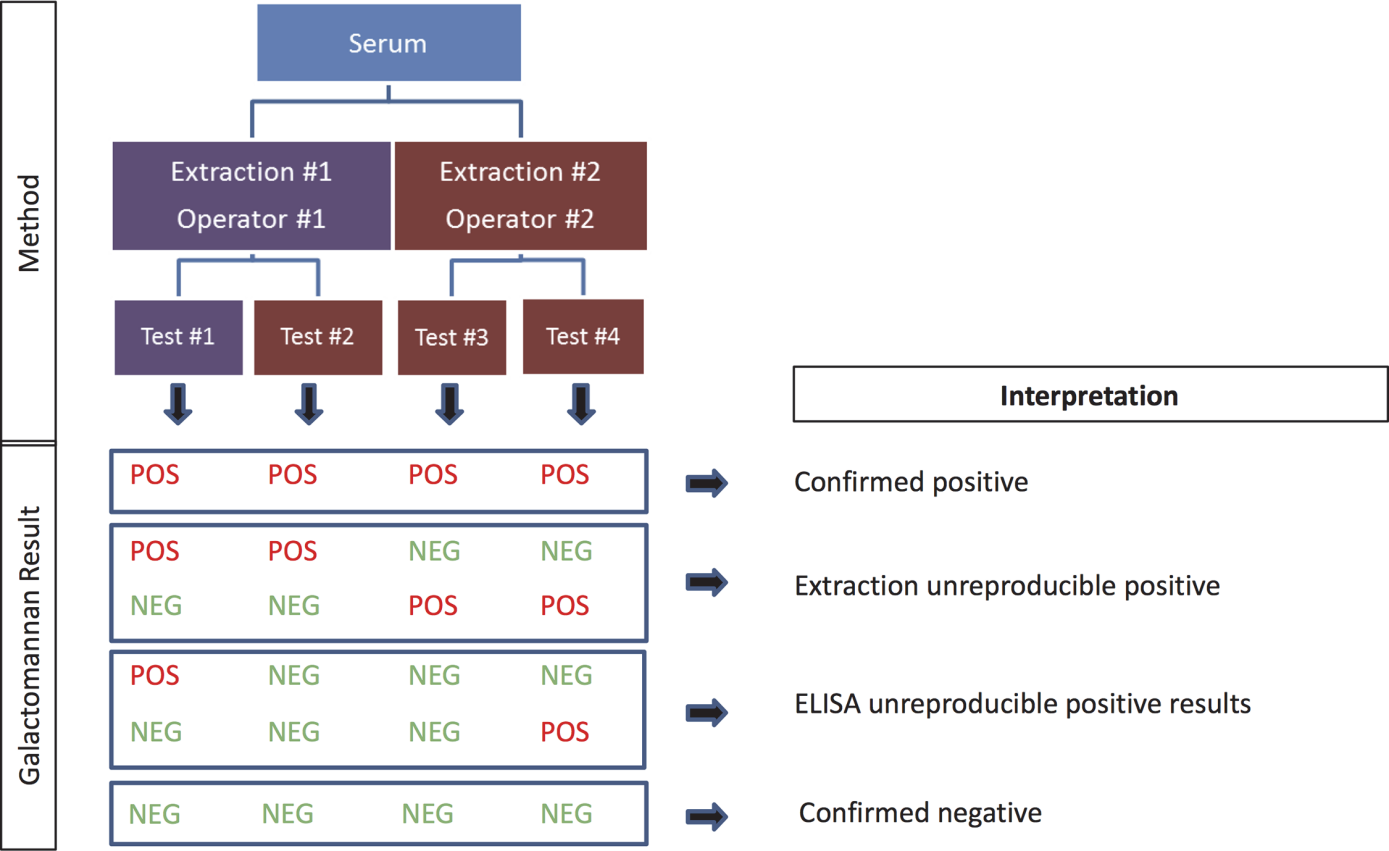
Schematic representation of the reproducibility study. Each serum was extracted twice by two different workers. Interpretation was as shown knowing that other combinations were possible but that only those obtained during the study are shown. Results close to the positivity threshold value (GM optical density index = 0.5) were classified as non-conclusive taking into account the interassay coefficient of variation of the kit.

We tested all the consecutive sera over 22 consecutive working days using the #62794 kit from February 17^th^ to March 10^th^ 2014. At day 0, each serum sample was tested four times (Fig 1). One test was performed by three different routine technicians (Operator#1 Test#1) over the study period without any change to the usual procedure. The three other tests were performed by a unique technician (Operator#2) blind of routine results; Test#2 was performed on the same GM extraction as Test#1, and Tests #2 and #3 on a different GM extraction.

At day 1, all the samples with at least one of the four tests (Test#1-#4) GM-ODI>0.5 were retested similarly as Day 0. Test#5 was performed by three different routine technicians (Operator#1 Extraction#3 Test#5) following the usual procedure. Three other tests were performed by Operator#2; Test#6 was performed on Extraction#3, and Tests #7 and #8 on Extraction#4.

The samples were classified as confirmed negative (Test#1–4 with ODI <0.5), confirmed positive (Test#1–4 with ODI ≥0.5) and unreproducible positive subdivided in extraction and ELISA unreproducible positive. Extraction unreproducible positive corresponded to a positivity linked to one of the two extractions (Test#1 and Test#2 ODIs ≥0.5, and Test#3 and Test#4 ODIs <0.5, or conversely). ELISA unreproducible positive corresponded to the positivity related to the ELISA performing with only one replicate with GM-ODI ≥0.5 (Fig 1). When at least one Test#1-#4 GM-ODI value was <0.5 but with the mean of the four tests within the 95% CI (0.36–0.64), these samples were classified as non-conclusive. A new classification was performed after retesting at Day 1 using the same criteria.

The remaining serum after the reproducibility study was stored at -20°C for 8 months. After thawing, a new GM extraction was performed and Test#9 performed by a unique routine technician in a single run. Test#9 was compared to Test#1.

### Statistical analyses

Statistical analyses were performed using the software online OpenEpi Software Version 2.3.1, Analyse-it SoftwareVersion 2.25, and Prism 5.0 (GraphPAD Software). We used Fisher's exact test or chi-squared test for categorical variables, and *t*-tests or one-way ANOVA for continuous variables with a normal distribution with p<0.05 significance.

## Results

We tested 567 sera from 235 patients over 22 consecutive working days in 16 series with the lot # 3L0024 of the kit. Seventeen samples were excluded from the reproducibility analysis since their volume did not allow all the tests.

Among the 550 remaining samples, 520 (94.5%) were confirmed negative upon Test#1-#4 results and 30 sera (5.5%) had at least one test positive (Table 1). Fifteen of these 30 sera (50%) were confirmed positive with upon Test#1-#4positive results, of which two (S203 and S 277, Table 1) were from one patient with probable invasive aspergillosis according to the European Organization for Research and Treatment of Cancer/ Mycoses Study Group [8].; 5 sera (16.7%) were non-conclusive; 10 sera (33.3%) were unreproducible positive divided in 6/10 (60%) extraction and 4/10 (40%) ELISA unreproducible positive (Table 1).

**Table 1:** Galactomannan optical density index (GM-ODI) results of the reproducibility study upon the four tests (Test#1-#4) of the serum samples performed at Day 0, the four tests (Test#5-#8) performed at Day 1, and the GM-ODI after 8 month storage at -20°C (Test#9).

	Day 0					Day 1						Month 8 after -20°C storage	
	Extraction#1		Extraction#2			Extraction#3		Extraction#4				Extraction#5	
Sample number	Test#1	Test#2	Test#3	Test#4	Day 0 classification	Test#5	Test#6	Test#7	Test#8	Day 1 classification	Test#5 ODI-Test#1 ODI	Test#9	Test#9 ODI-Test#1 ODI
S120	6.00	6.00	6.00	6.00	Confirmed Positive	6.00	6.00	6.00	6.00	Confirmed Positive	0.00	6.00	0.00
S325	6.00	6.00	6.00	6.00	Confirmed Positive	6.00	6.00	6.00	6.00	Confirmed Positive	0.00	6.00	0.00
S522 ^**a**^	6.00	6.00	6.00	6.00	Confirmed Positive	6.00	6.00	6.00	6.00	Confirmed Positive	0.00	6.00	0.00
S203	1.27	1.27	1.20	1.27	Confirmed Positive	1.17	1.10	1.27	1.18	Confirmed Positive	-0.10	1.11	-0.14
S373	0.81	0.85	0.78	0.89	Confirmed Positive	0.91	0.90	0.85	0.89	Confirmed Positive	0.10	0.93	0.10
S575	0.78	0.84	0.73	0.75	Confirmed Positive	0.84	0.96	0.84	0.80	Confirmed Positive	0.06	Not available	Not available
S72	0.71	0.71	0.65	0.62	Confirmed Positive	0.89	0.91	0.71	0.75	Confirmed Positive	0.19	0.75	0.04
S366	0.62	0.59	0.56	0.67	Confirmed Positive	0.67	0.64	0.60	0.67	Confirmed Positive	0.05	0.71	0.09
S469	0.60	0.58	0.50	0.53	Confirmed Positive	0.62	0.61	0.61	0.56	Confirmed Positive	0.02	0.51	-0.04
S568	0.69	0.68	0.64	0.57	Confirmed Positive	0.62	0.59	0.54	0.62	Confirmed Positive	-0.07	0.62	-0.07
S208	0.67	0.77	0.65	0.77	Confirmed Positive	0.61	0.67	0.62	0.64	Confirmed Positive	-0.06	0.65	-0.02
S272	1.16	1.12	1.36	1.55	Confirmed Positive	1.26	1.07	1.19	0.99	Confirmed Positive	0.10	1.35	0.19
S277	0.93	0.84	1.05	1.20	Confirmed Positive	1.01	0.88	0.79	0.91	Confirmed Positive	0.08	0.87	-0.10
S262	0.54	0.53	0.60	0.73	Confirmed Positive	0.69	0.66	0.67	0.64	Confirmed Positive	0.15	Not available	Not available
S44	1.10	0.65	0.64	0.91	Confirmed Positive	0.79	0.72	0.80	0.71	Confirmed Positive	-0.31	0.60	-0.5
S194	0.54	0.57	0.44	0.55	Non-Conclusive	0.48	0.43	0.58	0.56	Non-Conclusive	-0.06	Not available	Not available
S311	0.58	0.58	0.48	0.65	Non-Conclusive	0.63	0.65	0.60	0.58	Non-Conclusive	0.05	0.60	0.02
S245	0.49	0.46	0.54	0.55	Non-Conclusive	0.50	0.46	0.43	0.41	Non-Conclusive	0.01	0.46	-0.03
S108	0.44	0.42	0.35	0.55	Non-Conclusive	0.46	0.47	0.43	0.45	Non-Conclusive	0.02	0.43	-0.01
S370	0.41	0.37	0.45	0.58	Non-Conclusive	0.53	0.53	0.53	0.39	Non-Conclusive	0.12	0.58	0.17
S519	0.47	0.48	1.10	1.14	Extraction Unreproducible Positive	0.11	0.09	0.09	0.11	Confirmed Negative	Not relevant	0.06	nr
S353 ^**a**^	0.60	0.57	0.07	0.07	Extraction Unreproducible Positive	0.09	0.10	0.07	0.08	Confirmed Negative	Not relevant	0.06	nr
S71	0.67	0.67	0.07	0.06	Extraction Unreproducible Positive	0.15	0.16	0.07	0.13	Confirmed Negative	Not relevant	0.07	nr
S77	3.24	3.05	0.07	0.08	Extraction Unreproducible Positive	0.12	0.16	0.09	0.11	Confirmed Negative	Not relevant	0.06	nr
S37	0.37	0.31	0.43	0.55	ELISA Unreproducible Positive	0.27	0.27	0.30	0.26	Confirmed Negative	Not relevant	0.23	nr
S176	0.32	0.44	0.44	0.74	ELISA Unreproducible Positive	0.36	0.29	0.37	0.43	Confirmed Negative	Not relevant	0.33	nr
S25	0.85	0.44	0.39	0.40	ELISA Unreproducible Positive	0.59	0.53	0.50	0.47	Non-Conclusive	Not relevant	0.44	nr
S86	0.70	0.20	0.10	0.08	ELISA Unreproducible Positive	0.16	0.18	0.14	0.06	Confirmed Negative	Not relevant	0.08	nr
S128	1.34	0.08	0.12	0.12	ELISA Unreproducible Positive	0.15	0.17	0.13	0.12	Confirmed Negative	Not relevant	0.12	nr
S265	6.00	0.08	0.11	0.13	ELISA Unreproducible Positive	0.09	0.10	0.08	0.13	Confirmed Negative	Not relevant	0.06	nr

^a ^Day 1 tests performed after 72h at 4°C.

The samples were classified as confirmed negative [all tests with GM optical density index (GM-ODI) <0.5], confirmed positive (all tests with GM-ODI ≥0.5), extraction unreproducible positive (Test#1 and Test#2 ODIs ≥0.5, and Test#3 and Test#4 GM-ODIs <0.5, or conversely), and ELISA unreproducible positive (only one test with GM-ODI ≥0.5). The same rules were applied at Day 1.

**Fig 2:**
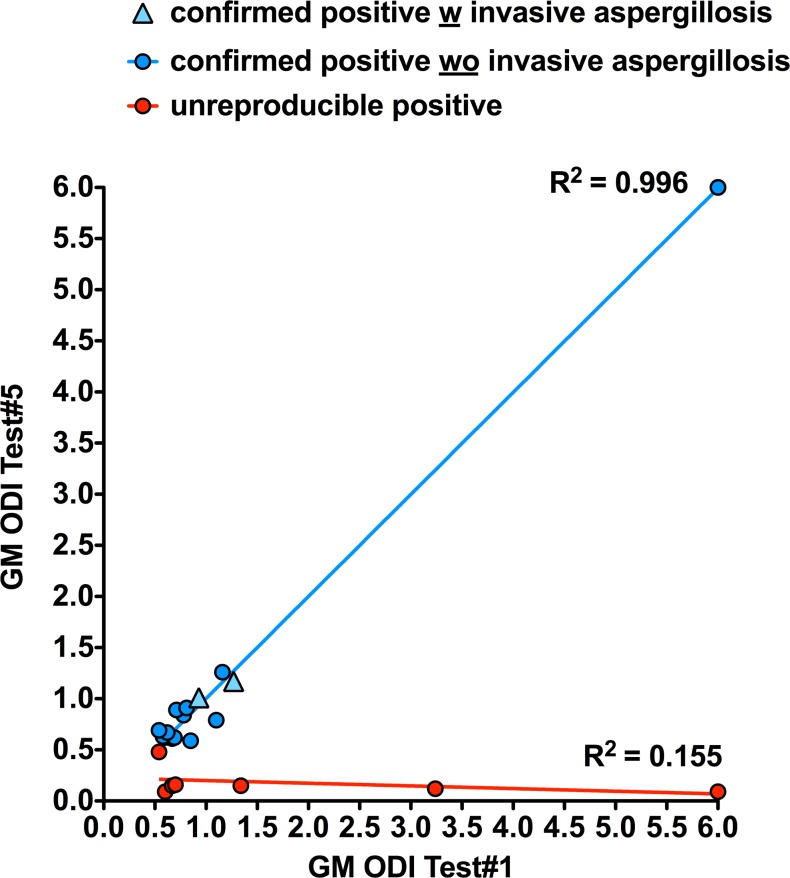
Graph plotting initial Test#1 against Test#5 (see Fig 1) performed after serum storage ≤72h at +4°C. A linear trend line was observed for confirmed positive and unreproducible positive results as in Fig 2.

When retested at Day 1 with four additional tests (Test#5–8) after storage at 4°C, the Day 0 confirmed positives and non-conclusives were similarly classified but for one non-conclusive which turned confirmed positive (S311 Table 1). For the Day 0 unreproducible positive results, all turned confirmed negative but for one which was classified as non-conclusive (S25 Table 1). Table 1 also shows Test#5 compared with Test#1 results after storage at 4°C for <72h as in our routine conditions. Test#5 results of the confirmed positives and non-conclusive were similar to Test#1 GM-ODIs (paired t-test: p = 0.49). Quantitatively, we observed two distinct populations when comparing Day 0 and Day 1 results. On one hand, the confirmed positives and non-conclusives with no GM-ODI variation (slope at 0.99). On the other hand, the ELISA and extraction unreproducible positives represented a distinct group of sera (Fig 2).

Among the 30 sera with at least one GM-ODI>0.5 at Day 0, 27 had enough serum left to be stored at -20°C which were retested (Extraction#5 Test#9) after 8 months with the lot # 4G1027 of the kit. Table 1 shows Test#9 in comparison with Test#1 results. Once again, the confirmed positive and non-conclusive sera had similar GM-ODI (paired t-test: p = 0.63) whereas the unreproducible positives at Day 0 were all retested negative as at Day1.

## Discussion

The present reproducibility study allowed us to clearly delineate on initial testing of the positive GM results between those which were confirmed positive and the unreproducible positive results. For the first time, we quantified the extraction and ELISA causes of these unreproducible positives, showing that both can intervene at a global rate of 33% (10/30), within the range of previous reports: 39% [16], 40% [14], 48% [12], 55% [18], and 64% [15] of the tested samples. Therefore, in contrast to hypotheses which favor a particular component and/or storage of the serum to explain the unreproducible positive results [13,15,16], we highlighted that most of them, if not all, were due to technical variation either during the extraction process or in the ELISA plate itself. Therefore two populations of sera can be differentiated; the first one with a positive result related to the sample for which the GM-ODI remains unchanged upon retesting, either after storage at 4°C for <72h or at—20°C for at least 8 months, and the second one with an initial positive result related to technical variation for which a second testing after storage at 4°C for <72h is negative.

These results lead us to reconsider all articles that have been recently published on GM reproducibility and stability during storage where these two populations were not identified [13,15,16,20–22]. In contrast to the present study, the previous studies performed only one test on first determination and one repeat after storage [13,15,16]. Consequently they could not distinguish the initial positivity related to samples from that related to any technical problem. Interestingly, a lot of the figures from these studies look like ours with two groups of sera. One group for which there is no or low difference between tests and the other one for which the samples completely turned negative. Therefore, the conclusion that freezing reduces the risk of a false positive result [15] cannot be maintained without confirming that the initial positivity is a confirmed positive. In contrast, some studies like ours showed little impact of long-term storage at—20°C on GM-ODI values and that storage did not affect the sera from patients with invasive aspergillosis [20]. One can hypothesize that sera from patients with invasive aspergillosis were confirmed positive, hence the lack of impact of freezing on GM-ODIs. Similarly, several reports [12,14,16,17] noticed that the samples that turned negative on repeated test corresponded to patients with a low probability of invasive aspergillosis, which is also consistent with our results.

Unfortunately, we could not ascribe unreproducible positive results to a specific procedure failure or to a specific technician. The manual processing of the test is very sensitive to small variations of the heat extraction procedure for instance. An external source independent to the operator can occur. One can suspect GM contamination from the water-bath, which can be regularly checked, or from tips or tubes for treatment, which could make the use of antigen-free material compulsory. The various origins can explain the different rates of unreproducible positive results between laboratories. Whatever the source of this lack of reproducibility, it strongly supports re-analysis of a positive sample as soon as possible [1]. Of course, to perform a second test on the same serum does not completely exclude the possibility to obtain a second unreproducible positive result. Therefore, obtaining serial samples in parallel is also recommended [1], acknowledging that the risk of false positive cannot be definitively excluded. The non-conclusive category we created for the reproducibility study is questionable, since we did not classify non-conclusive sera with all GM tests positive but within the CV of the GM assay. However, in case of possible false positive results and non-conclusive results, given the risk of misidentifying an invasive aspergillosis, to provide a positive answer to the clinicians seems preferable. That is why we have not created a non-conclusive category in our routine procedure and insist on the GM-ODI kinetics upon serial samples for final correct interpretation.

The present work was focused on the analytical performances of the Platelia *Aspergillus* Ag kit and was not designed for deciphering between false positives due to an exogenous source of GM in serum as a consequence of blood contamination with antibiotics [23] or of other invasive fungal infections such as histoplasmosis, cryptococcosis, or penicilliosis [24]. Nor do we address the issue of false positive of digestive origin [10,25]. We think that confirmed GM positives, as a general rule, must be interpreted with care using all the information needed to make a final diagnosis of invasive aspergillosis.

In conclusion, the reasons why some sera turned totally negative on retesting are operator-dependent. We also showed the good stability of GM for at least 72h at +4°C and over 8 months at -20°C when dealing with the confirmed positive sera only. As a consequence, analytical false positive results must be eliminated in performing a second evaluation of the sample as soon as possible to avoid useless investigations or treatments and for correct interpretation when re-testing after storage. Further research on unreproducible positive results is warranted to decrease their occurrence, knowing that full automation of the assay is unlikely for technical reasons.

## Supporting Information

S1 Data Table S1 _data_Table.xls. (XLSX)
